# DIETARY BEHAVIORS OF AFRICAN IMMIGRANTS LIVING WITH DIABETES AND/OR HYPERTENSION: A QUALITATIVE STUDY

**DOI:** 10.1093/geroni/igae098.3603

**Published:** 2024-12-31

**Authors:** Onome Osokpo, Lisa Lewis, Danny Bracy, Dolapo Adeniji, Ayomide Bankole, Barbara Riegel

**Affiliations:** University of Illinois Chicago, Chicago, Illinois, United States; Rutgers, The State University of New Jersey, Camden, New Jersey, United States; University of Pennsylvania, Philadelphia, Pennsylvania, United States; Indiana State University, Avon, Indiana, United States; Duke University, Durham, North Carolina, United States; University of Pennsylvania, Philadelphia, Pennsylvania, United States

## Abstract

Healthy dietary behaviors (e.g., low-salt diet) are a critical element of self-care and the cornerstone in managing diabetes and/or hypertension. Social determinants (e.g., cultural preferences and social support) influence dietary behaviors. However, little is known about the influence of social determinants on dietary behaviors of African immigrants living with diabetes and/or hypertension accounting for their level of acculturation. We conducted a descriptive qualitative study of a purposive sample of 24 self-identified sub-Saharan African immigrant adults in the Northeastern U.S. with symptomatic hypertension and/or diabetes who spoke and understood English. Based on scores from the Short Acculturation Scale, African immigrants were classified as more or less acculturated. The sample was mainly Nigerian (75%) and males (67%), with a mean age of 59 years [40-72]. The sample was highly educated, acculturated, employed, and reported having enough income to meet their needs. Compared to more-acculturated immigrants, less-acculturated immigrants regularly ate more African or “traditional food,” vegetables and rice, and avoided salt and fatty/fast/processed American foods. Less-acculturated immigrants cooked their meals and learned their diets from previous education or their workplace. Compared to the less acculturated, more acculturated participants ate fruits regularly and avoided meat or red meat. Interestingly, the more acculturated participants reported learning about their diet from family and friends and noted that family members cook the meals in their household. Interventions to promote healthy diets among immigrants should take into consideration cultural dietary preferences, influence of family, friends, and non-familial social connections, and level of acculturation.

